# Utility of transesophageal echocardiography in the ICU: a preliminary US perspective

**DOI:** 10.1186/cc12120

**Published:** 2013-03-19

**Authors:** A Kaynar, D Phillips, H Gomez, M Lischner, S Melhem, K Subramaniam, M Pinsky

**Affiliations:** 1 University of Pittsburgh, PA, USA

## Introduction

While TEE is providing a direct assessment of the cardiac function and volume status as a diagnostic tool, until recently it has been impractical to be continuously available for monitoring. A new disposable, monoplanar TEE probe (ImaCor) can remain in the patient for up to 72 hours, allowing repeated measures of ventricular function and volume status, parameters needed to monitor response to therapy.

## Methods

We assessed the benefit these TEE data provided in the assessment of five domains: hypovolemia, right ventricular dysfunction, left ventricular dysfunction, sepsis, and valvular abnormality. Bedside practitioners listed their diagnoses before and after seeing primary TEE images perform by trained physicians. We used a 0 to 5 Likert scale to assess differential diagnosis before and after the TEE, comparing changes using a paired *t *test.

## Results

**Figure 1:**
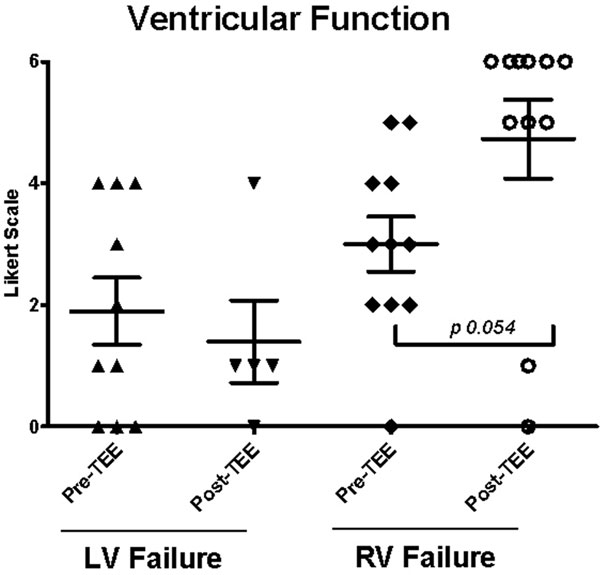
**Ventricular function according to the Likert scale**.

**Figure 2:**
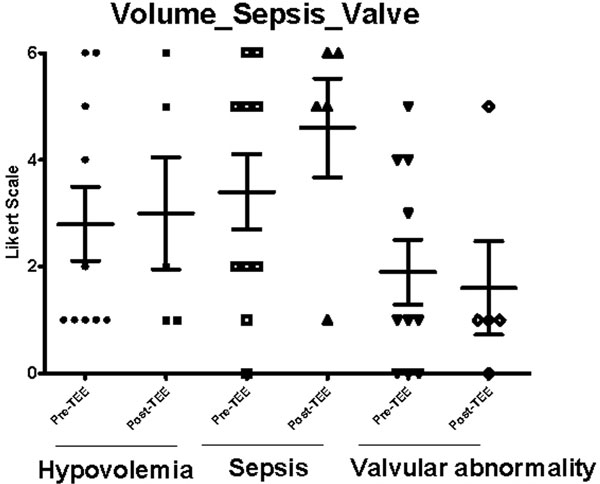
**Volume status, valve, sepsis according to the Likert scale**.

All requests for TEE were to access hemodynamic instability. A total of 18 patients were screened and nine were eligible, in which 16 total TEE studies were performed. There were no complications with TEE and all patients tolerated the long-term placement of the probe well. Of the five diagnostic domains studied, right ventricular failure was the most commonly underdiagnosed contributor to the hemodynamic instability among patients prior to TEE (*P *= 0.054) (Figures 1 and 2).

## Conclusion

Our results suggest that having continuously available TEE for monitoring and management of hemodynamically unstable patients increases awareness of right ventricular dysfunction in the ICU.

## References

[B1] Vieillard-Baron A Intensive Care Med 2004 30 1734 1739 15375649 10.1007/s00134-004-2361-y

[B2] Monnet X Intensive Care Med 2005 31 1195 1201 10.1007/s00134-005-2731-0 16059723

